# Trends in colorectal cancer mortality in China from 2004 to 2021: an epidemiological analysis based on national disease surveillance data

**DOI:** 10.3389/fonc.2026.1780470

**Published:** 2026-05-01

**Authors:** Yuting Yang, Minghao Chen, Aowen Chen, Changquan Huang

**Affiliations:** Department of Clinical Research, Sichuan University Affiliated Chengdu Second People’s Hospital, Chengdu Second People’s Hospital, West China School of Medicine, Sichuan University, Chengdu, Sichuan, China

**Keywords:** China, colorectal cancer, disparity, epidemiological trend, mortality

## Abstract

**Background:**

Colorectal cancer (CRC) is a common malignant tumor in the digestive system worldwide. In China, there are significant challenges in prevention and control, and there is a lack of relevant evidence regarding the long-term epidemiological trends. This study aims to evaluate the CRC mortality data in China from 2004 to 2021 and to explore the trends of age-specific and stratified mortality rates by age, gender, urban-rural areas, and regions.

**Method:**

The CRC mortality data from 2004 to 2021 were extracted from the China Disease Surveillance Point (DSP) system. After excluding low-quality data, the mortality rates for five age groups, gender, urban-rural residence, and regions were calculated. Independent sample t-tests and 95% confidence intervals (CI) were used to compare differences between groups and the average mortality rates before and after 2012. The long-term trend was analyzed using the age-period-cohort (APC) model and the analysis was conducted using R 3.1.2 and SAS 9.4.

**Result:**

The standardized CRC mortality rate for Chinese adults was 11.5 per 100,000 (95% CI: 11.3 - 11.7), showing a significant age gradient. The mortality rate for men (14.2 per 100,000) was higher than that for women (9.1 per 100,000), and the mortality rate in urban areas (14.1 per 100,000) was higher than that in rural areas (10.0 per 100,000). The overall mortality rate remained stable (average annual change rate -0.10%), but the mortality rate for people aged 65 and above significantly increased, especially for those aged 85 and above (average annual change rate 2.52%, 95% CI: 0.98 - 4.09). The mortality rate increase for men aged 85 and above in the western rural areas was the most significant (average annual change rate 5.53%, 95% CI: 3.14 - 7.97).

**Conclusion:**

The mortality rate of CRC in China shows significant differences among populations and regions. The burden is particularly heavy for men, urban residents, and people in the eastern coastal areas. Strengthening early screening for high-risk groups and optimizing the allocation of regional medical resources are crucial for reducing the burden of CRC.

## 1 Introduction

CRC is the fourth leading cause of cancer-related deaths globally (1) and ranks fourth among cancer deaths in China (2). As of 2019, Chinese patients accounted for 30% of global CRC cases. The high cost of CRC treatment imposes a heavy economic burden on society (3).

Previous studies (4–7) have shown significant regional, urban-rural, and sex-based disparities in CRC incidence and mortality. Unlike the stabilizing or declining CRC trends observed in highly developed countries, the disease burden continues to rise in developing nations, including China. Although China’s “Healthy China 2030” strategy has incorporated CRC into free urban cancer screening programs (8), achieving a 5-year survival rate of 56.9% (9), national mortality rates have not significantly declined—indicating new challenges for CRC control and prevention in China.

Existing studies on CRC mortality in China are mostly limited to single regions or short-term trend analyses. Comprehensive, nationwide, long-term, and multidimensional studies are lacking. Therefore, this study aims to analyze long-term trends in CRC mortality using DSP data from 2004 to 2021, with a focus on differences by age, sex, urban-rural residence, and regional economic development. The findings are expected to improve the understanding of CRC epidemiology in China and provide a scientific basis for targeted prevention strategies.

## 2 Methods

### 2.1 Data sources

The CRC mortality data from 2004 to 2021 were extracted from the China Disease Surveillance Point (DSP) system. The DSP system covers surveillance sites across all 31 provincial-level administrative regions in China and represents approximately 23–24% of the national population.

Urban–rural classification was based on the administrative designation of each surveillance site according to national statistical standards. Regional classification followed the official stratification used in the China Cause of Death Surveillance system, in which provinces were grouped into eastern, central, and western regions.

In this study, the outcome was cause-specific mortality from CRC, defined as deaths with underlying causes coded as ICD-10 C18–C21. Surveillance sites with an average underreporting rate exceeding 10% or with missing rates of key variables greater than 15% were excluded.

The age-standardized mortality rates (ASMRs) were obtained directly from the China Cause-of-Death Surveillance Dataset (2021), in which rates were calculated using the direct standardization method based on the age structure of the 2000 and 2010 Chinese national census populations. ASMR was calculated as:


$$ASMR\;=\;\frac{\sum nPx\;\cdot \;nMx}{\sum nPx}$$

The population structure of each year was used as the denominator. The analysis included adults aged ≥20 years, and stratified analyses were conducted by sex, urban–rural residence, region, and 5-year age groups.

Both classifications were predefined within the DSP system and were not redefined in the present study.

### 2.2 Statistical analysis

Age-specific mortality rates were calculated by sex, urban–rural residence, region, and age group. Independent-sample t-tests were used to compare average mortality rates between men and women, and between urban and rural populations. Differences in mortality rates between 2004–2007 and 2018–2021 were evaluated using mean values and 95% confidence intervals (CIs).

Age–period–cohort (APC) models (10) were used to assess the independent effects of age, calendar period, and birth cohort on mortality trends. The APC model assumes a log-linear Poisson regression structure for age-specific mortality rates. Because age, period, and cohort are linearly dependent (cohort = period − age), the identification problem was addressed using standard APC modeling procedures implemented in R. All statistical analyses were conducted using R version 3.1.2 and SAS version 9.4.

## 3 Results

### 3.1 CRC mortality by sex, area, and region, China, 2004–2021

**Table 1** Colorectal cancer mortality by sex, area, and region, China, 2004–2021. Among adults aged 20 and above, the total number of monitored individuals was 2,336,766,580, and the total number of deaths due to CRC was 317,494. After standardization, the overall standardized mortality rate of CRC was 11.5 (11.3 - 11.7), meaning that for every 100,000 people, 11.5 died of CRC. Stratified by sex, the mortality rate was significantly higher in males [14.2 (13.9–14.6)] than in females [9.1 (8.9–9.3)]. Urban residents had a higher mortality rate [14.1 (13.8–14.4)] compared to rural residents [10.0 (9.7–10.4)]. By region, the highest mortality rate was observed in the eastern region [12.5 (12.2–12.7)], followed by the central [11.0 (10.5–11.4)] and western regions [10.5 (10.1–11.0)]. Age-specific analysis showed a marked increase in mortality with advancing age: Ages 20–44: 1.2 (1.2–1.3), Ages 45–59: 8.2 (7.9–8.4), Ages 60–74: 32.7 (31.9–33.5), Ages 75–84: 88.0 (85.6–90.5), Ages ≥85: 152.2 (133.3–171.1). This trend remained consistent across sex and urban-rural subgroups.These findings suggest that although the overall CRC mortality rate is relatively stable, there is a clear upward trend in specific subpopulations, with the highest burden observed among males, urban residents, individuals in the eastern region, and particularly those aged 85 years and above.

**Table 1:** Colorectal cancer mortality by sex, area, and region, China, 2004–2021.

		Survey		Colorectal cancer deaths			
Age	Subgroup type	Population	*Percentage	Population	*Percentage	Age-adjusted mortality	95% CI
Aged all (≥20 years)		2,336,766,580	100.0%	317,494	100.0%	11.5	(11.3-11.7)
Sex	Men	1,172,864,380	50.2%	188,115	59.2%	14.2	(13.9-14.6)
	Women	1,163,902,200	49.8%	129,379	40.8%	9.1	(8.9-9.3)
Area	Urban	825,015,563	35.3%	133,037	41.9%	14.1	(13.8-14.4)
	Rural	1,511,751,017	64.7%	184,457	58.1%	10.0	(9.7-10.4)
Region	Eastern region	936,175,171	40.1%	148,522	46.8%	12.5	(12.2-12.7)
	Central region	807,062,309	34.5%	96,467	30.4%	11.0	(10.5-11.4)
	Western region	593,529,100	25.4%	72,505	22.8%	10.5	(10.1-11.0)
Aged 20-44		1,153,487,773	49.4%	13,619	4.3%	1.2	(1.2-1.3)
Sex	Men	584,995,185	25.0%	7,679	2.4%	1.4	(1.3-1.4)
	Women	568,492,588	24.3%	5,940	1.9%	1.1	(1.0-1.2)
Area	Urban	411,345,084	17.6%	4,480	1.4%	1.2	(1.1-1.3)
	Rural	742,142,689	31.8%	9,139	2.9%	1.3	(1.2-1.3)
Region	Eastern region	445,321,407	19.1%	5,110	1.6%	1.2	(1.1-1.2)
	Central region	404,543,424	17.3%	4,682	1.5%	1.2	(1.1-1.3)
	Western region	303,622,942	13.0%	3,827	1.2%	1.3	(1.2-1.4)
Aged 45-59		687,892,518	29.4%	54,037	17.0%	8.2	(7.9-8.4)
Sex	Men	347,391,918	14.9%	33,119	10.4%	9.8	(9.5-10.1)
	Women	340,500,600	14.6%	20,918	6.6%	6.5	(6.2-6.8)
Area	Urban	244,690,976	10.5%	20,722	6.5%	9.0	(8.5-9.5)
	Rural	443,201,542	19.0%	33,315	10.5%	7.7	(7.4-7.9)
Region	Eastern region	282,455,074	12.1%	22,921	7.2%	8.3	(8.0-8.6)
	Central region	236,781,689	10.1%	17,971	5.7%	8.2	(7.7-8.6)
	Western region	168,655,755	7.2%	13,145	4.1%	8.0	(7.6-8.3)
Aged 60-74		368,733,994	15.8%	122,800	38.7%	32.7	(31.9-33.5)
Sex	Men	184,059,764	7.9%	77,322	24.4%	40.7	(39.4-42.0)
	Women	184,674,230	7.9%	45,478	14.3%	24.9	(24.2-25.5)
Area	Urban	124,499,964	5.3%	49,240	15.5%	39.4	(38.2-40.5)
	Rural	244,234,030	10.5%	73,560	23.2%	29.0	(28.1-30.0)
Region	Eastern region	151,708,018	6.5%	54,911	17.3%	35.2	(34.1-36.4)
	Central region	124,978,462	5.3%	38,735	12.2%	31.5	(30.6-32.4)
	Western region	92,047,514	3.9%	29,154	9.2%	30.4	(29.2-31.6)
Aged 75-84		104,499,116	4.5%	92,784	29.2%	88.0	(85.6-90.5)
Sex	Men	47,846,717	2.0%	52,872	16.7%	111.0	(107.4-114.5)
	Women	56,652,399	2.4%	39,912	12.6%	69.3	(67.3-71.2)
Area	Urban	36,823,121	1.6%	40,984	12.9%	114.1	(110.0-118.2)
	Rural	67,675,995	2.9%	51,800	16.3%	72.7	(68.7-76.7)
Region	Eastern region	46,351,304	2.0%	45,714	14.4%	98.3	(95.3-101.2)
	Central region	33,814,922	1.4%	27,088	8.5%	82.7	(79.0-86.4)
	Western region	24,332,890	1.0%	19,982	6.3%	76.2	(70.8-81.6)
Aged 85 and above		22,153,179	0.9%	34,254	10.8%	152.2	(133.3-171.1)
Sex	Men	8,570,796	0.4%	17,123	5.4%	197.8	(171.4-224.2)
	Women	13,582,383	0.6%	17,131	5.4%	124.4	(109.2-139.5)
Area	Urban	7,656,418	0.3%	17,611	5.5%	225.5	(199.2-251.8)
	Rural	14,496,761	0.6%	16,643	5.2%	110.5	(95.9-125.1)
Region	Eastern region	10,339,368	0.4%	19,866	6.3%	183.8	(161.9-205.7)
	Central region	6,943,812	0.3%	7,991	2.5%	128.7	(109.7-147.7)
	Western region	4,869,999	0.2%	6,397	2.0%	121.1	(100.7-141.4)

Values are aggregated for the study period 2004–2021.

### 3.2 Differences in mortality rates by sex and urban-rural residence

**Figure 1:**
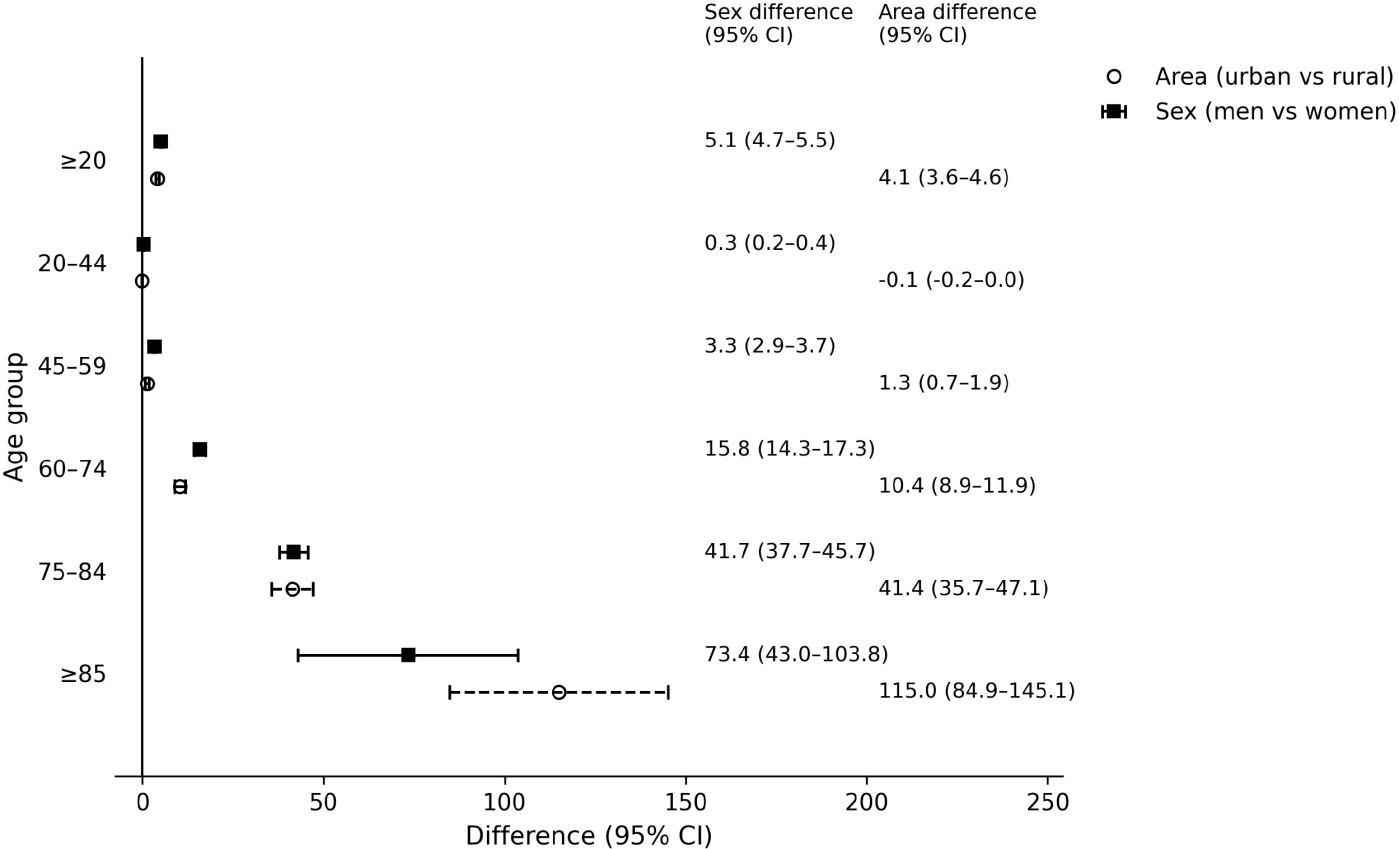
Absolute changes in colorectal cancer mortality rates between urban and rural areas and between men and women in China from 2004 to 2021.

**Table 2** and **Figure 1** illustrates the differences in CRC mortality rates between men and women as well as between urban and rural areas. In terms of sex differences, the overall CRC mortality rate among males was 5.1 per 100,000 higher than that of females, with a 95% confidence interval (CI) of 4.7–5.5. This gap widened progressively with age, reaching the largest difference of 73.4 (95% CI: 43.0–103.8) in individuals aged 85 years and above. Regarding urban-rural differences, the overall CRC mortality rate in urban areas was 4.1 per 100,000 higher than in rural areas (95% CI: 3.6–4.6). Similarly, this disparity increased markedly with age, peaking at a difference of 115.0 (95% CI: 84.9–145.1) in the 85+ age group.

**Table 2:** The absolute changes in age-standardized mortality rates of colorectal cancer in China between 2004 and 2021, both within urban and rural areas and between men and women.

Category	Age group	Difference	95% CI
Sex (Men vs Women)	all (≥20 years)	5.1	4.7–5.5
	20–44	0.3	0.2–0.4
	45–59	3.3	2.9–3.7
	60–74	15.8	14.3–17.3
	75–84	41.7	37.7–45.7
	≥85	73.4	43.0–103.8
Area (Urban vs Rural)	all (≥20 years)	4.1	3.6–4.6
	20–44	−0.1	−0.2–0.0
	45–59	1.3	0.7–1.9
	60–74	10.4	8.9–11.9
	75–84	41.4	35.7–47.1
	≥85	115	84.9–145.1

### 3.3 Changes in CRC mortality between two 4-year periods

**Figure 2:**
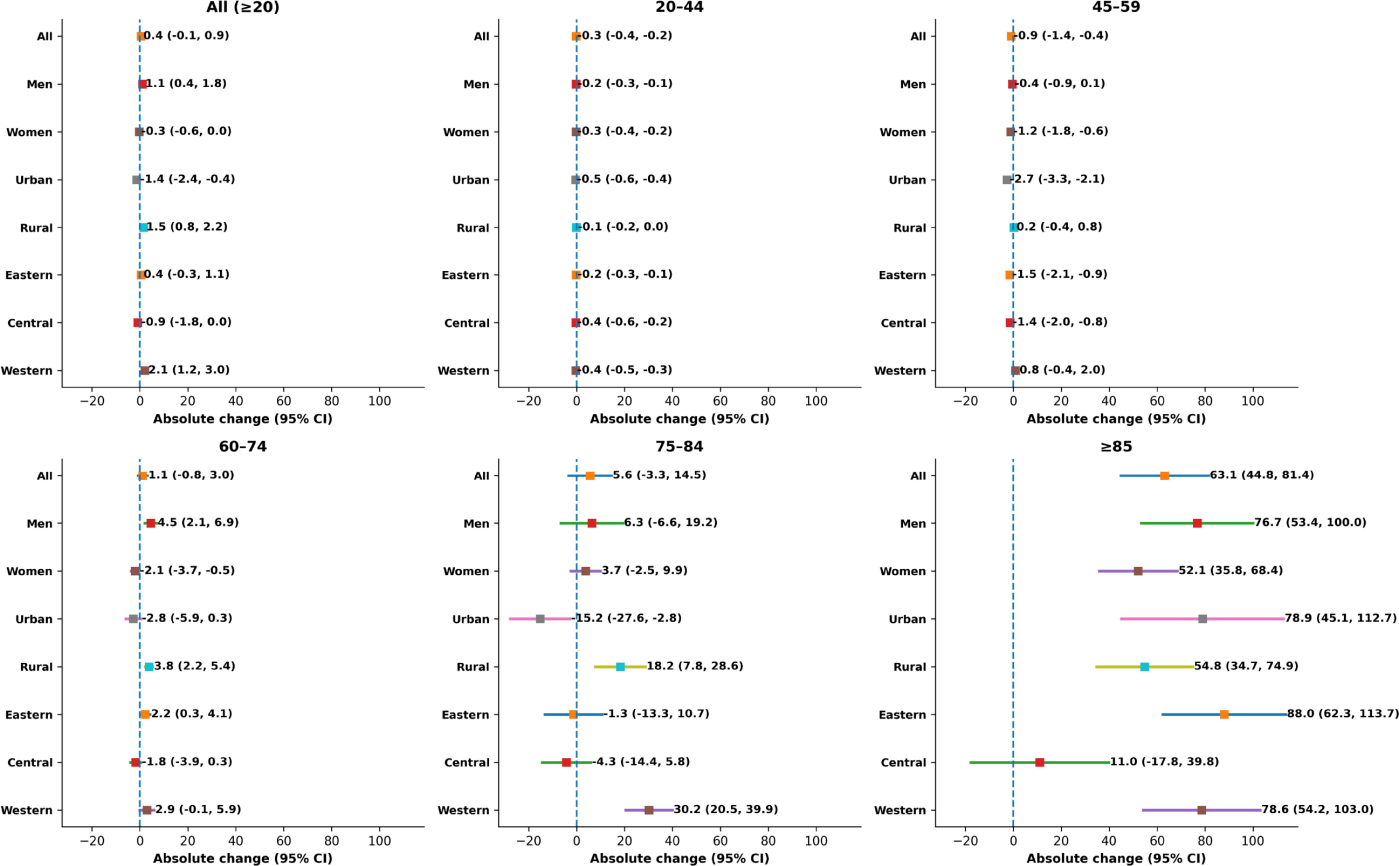
The absolute changes in age-standardized mortality rates of colorectal cancer in China from 2018 to 2021 (per 100,000 people) compared with those from 2004 to 2007.

**Table 3** and **Figure 2** depicts the changes in CRC mortality rates from 2018 to 2021 compared with those from 2004 to 2007, which were four years before. The CRC mortality rate has shown a significant upward trend. While no significant differences were observed across all age groups combined, mortality among individuals aged 85 years and above increased sharply by 63.1 per 100,000 (95% CI: 44.8–81.4). Among males, the overall CRC mortality rate increased by 1.1 per 100,000 (95% CI: 0.4–1.8). Significant increases were observed in men aged 60–74 and those aged 85 and above, with the latter showing the most rapid rise of 76.7 per 100,000 (95% CI: 53.4–100.0). Although the overall CRC mortality rate in females showed a declining trend, women aged 85 and above experienced the largest increase of 52.1 per 100,000 (95% CI: 35.8–68.4). In urban areas, despite a general downward trend, the mortality rate among residents aged 85 and above rose markedly by 78.9 per 100,000 (95% CI: 45.1–112.7). In rural areas, the overall CRC mortality increased by 1.5 per 100,000 (95% CI: 0.8–2.2), with residents aged 60 and above showing a consistent upward trend. The most substantial increase was observed among those aged 85 and above, rising by 54.8 per 100,000 (95% CI: 34.7–74.9). The western region recorded the highest increase across all regions, with an overall rise of 2.1 per 100,000 (95% CI: 1.2–3.0). The increase was most pronounced among individuals aged 75 and above, peaking at 78.6 per 100,000 (95% CI: 54.2–103.0) in the 85+ age group. These findings indicate a particularly significant rise in CRC mortality in recent years among older adults, males, and residents of rural and western regions.

**Table 3:** The absolute changes in age-standardized mortality rates of colorectal cancer in China from 2018 to 2021 (per 100,000 people) compared with those from 2004 to 2007.

Age group	Population subgroup	Absolute change (95% CI)
All (≥20)	All	0.4 (−0.1 to 0.9)
All (≥20)	Men	1.1 (0.4 to 1.8)
All (≥20)	Women	−0.3 (−0.6 to 0.0)
All (≥20)	Urban	−1.4 (−2.4 to −0.4)
All (≥20)	Rural	1.5 (0.8 to 2.2)
All (≥20)	Eastern region	0.4 (−0.3 to 1.1)
All (≥20)	Central region	−0.9 (−1.8 to 0.0)
All (≥20)	Western region	2.1 (1.2 to 3.0)
20–44	All	−0.3 (−0.4 to −0.2)
20–44	Men	−0.2 (−0.3 to −0.1)
20–44	Women	−0.3 (−0.4 to −0.2)
20–44	Urban	−0.5 (−0.6 to −0.4)
20–44	Rural	−0.1 (−0.2 to 0.0)
20–44	Eastern region	−0.2 (−0.3 to −0.1)
20–44	Central region	−0.4 (−0.6 to −0.2)
20–44	Western region	−0.4 (−0.5 to −0.3)
45–59	All	−0.9 (−1.4 to −0.4)
45–59	Men	−0.4 (−0.9 to 0.1)
45–59	Women	−1.2 (−1.8 to −0.6)
45–59	Urban	−2.7 (−3.3 to −2.1)
45–59	Rural	0.2 (−0.4 to 0.8)
45–59	Eastern region	−1.5 (−2.1 to −0.9)
45–59	Central region	−1.4 (−2.0 to −0.8)
45–59	Western region	0.8 (−0.4 to 2.0)
60–74	All	1.1 (−0.8 to 3.0)
60–74	Men	4.5 (2.1 to 6.9)
60–74	Women	−2.1 (−3.7 to −0.5)
60–74	Urban	−2.8 (−5.9 to 0.3)
60–74	Rural	3.8 (2.2 to 5.4)
60–74	Eastern region	2.2 (0.3 to 4.1)
60–74	Central region	−1.8 (−3.9 to 0.3)
60–74	Western region	2.9 (−0.1 to 5.9)
75–84	All	5.6 (−3.3 to 14.5)
75–84	Men	6.3 (−6.6 to 19.2)
75–84	Women	3.7 (−2.5 to 9.9)
75–84	Urban	−15.2 (−27.6 to −2.8)
75–84	Rural	18.2 (7.8 to 28.6)
75–84	Eastern region	−1.3 (−13.3 to 10.7)
75–84	Central region	−4.3 (−14.4 to 5.8)
75–84	Western region	30.2 (20.5 to 39.9)
≥85	All	63.1 (44.8 to 81.4)
≥85	Men	76.7 (53.4 to 100.0)
≥85	Women	52.1 (35.8 to 68.4)
≥85	Urban	78.9 (45.1 to 112.7)
≥85	Rural	54.8 (34.7 to 74.9)
≥85	Eastern region	88.0 (62.3 to 113.7)
≥85	Central region	11.0 (−17.8 to 39.8)
≥85	Western region	78.6 (54.2 to 103.0)

### 3.4 Changes in annual percent change in mortality rates in 2018

**Figure 3:**
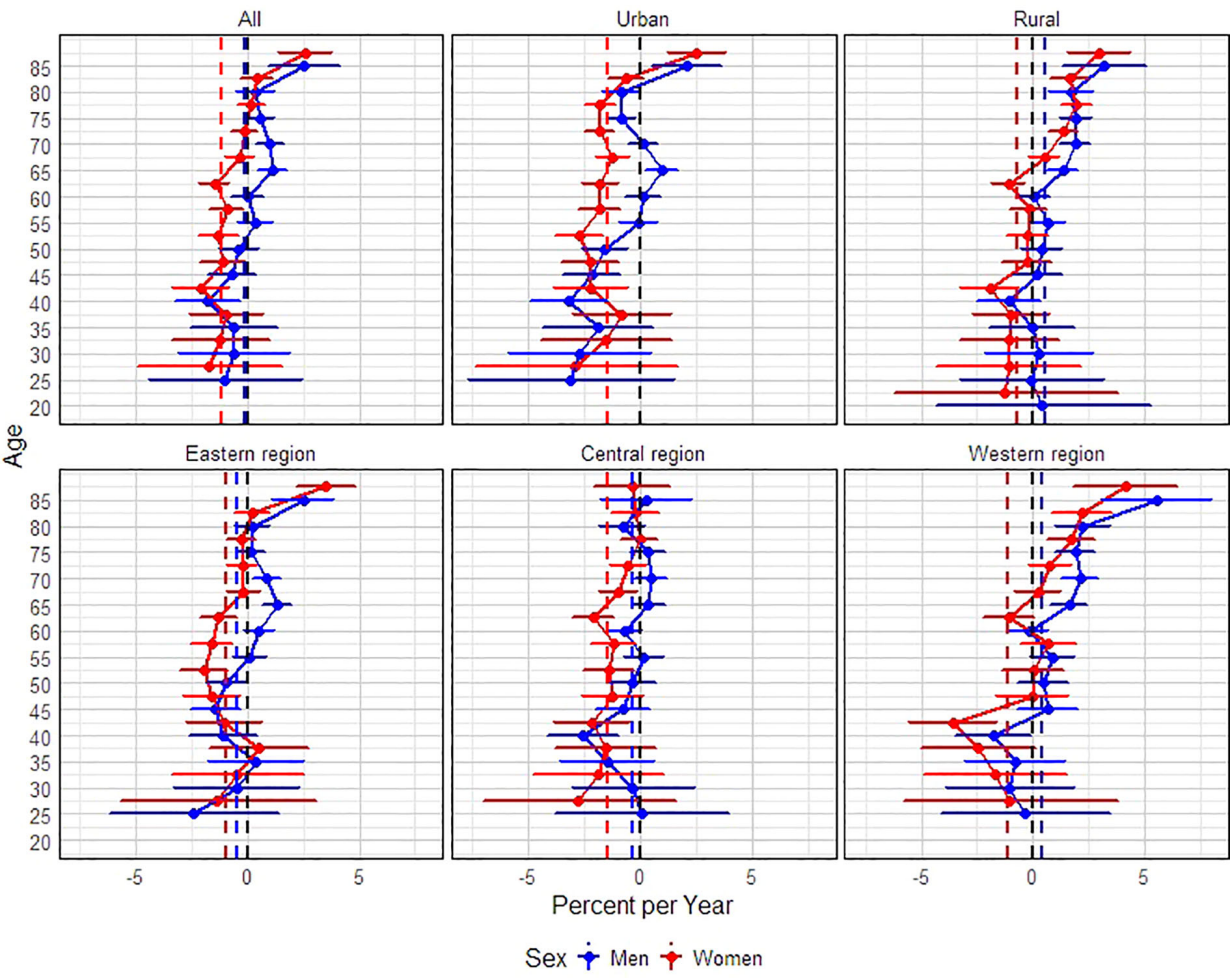
Projected annual percentage changes (APC) in colorectal cancer mortality rates by gender (men and women), urban and rural areas, and eastern, central and western regions.

**Table 4** and **Figure 3** depicts the changes in the APC mortality rate over the past 18 years. From 2004 to 2021, the age-standardized mortality rate (ASMR) of CRC among adult males aged 20 years and above remained relatively stable overall, with no significant upward or downward trend. However, age-specific analysis revealed a consistent annual increase in mortality among men aged 65–74 and those aged 85 and above. The most significant rise was observed in men aged 85 and above, with an APC of 2.52% (95% CI: 0.98–4.09%). This overall trend was mainly driven by increases in urban males aged 65–69 and those aged 85 and above. The most notable rise was seen in urban males aged 85 and above, with an APC of 2.05% (95% CI: 0.54–3.58%). In rural areas, men aged 85 and above also experienced a consistent annual increase, with the highest APC reaching 3.20% (95% CI: 1.37–5.08%). Regionally, males in the eastern region aged 65–74 and 85+ showed a rising trend in mortality, with the highest increase among those aged 85 and above, at 2.50% (95% CI: 1.16–3.85%). No significant changes were observed in the central region. In contrast, the western region showed a consistent upward trend in CRC mortality among men aged 65 and above, with the most pronounced increase seen in those aged 85 and above (APC: 5.53%, 95% CI: 3.14–7.97%).

**Table 4:** From 2004 to 2021, by urban and rural areas, the projected annual percentage changes (APC) of colorectal cancer mortality rates for different age groups and genders in China.

Region	Sex	Age (years)	Percent change (95% CI)
All	Men	20-24	-0.09 (-4.98 to 5.05)
All	Men	25-29	-1.01 (-4.35 to 2.45)
All	Men	30-34	-0.63 (-3.08 to 1.87)
All	Men	35-39	-0.62 (-2.52 to 1.32)
All	Men	40-44	-1.78 (-3.18 to -0.37)
All	Men	45-49	-0.69 (-1.76 to 0.39)
All	Men	50-54	-0.38 (-1.25 to 0.49)
All	Men	55-59	0.34 (-0.41 to 1.1)
All	Men	60-64	0.01 (-0.67 to 0.69)
All	Men	65-69	1.1 (0.48 to 1.73)
All	Men	70-74	1.02 (0.43 to 1.62)
All	Men	75-79	0.59 (-0.04 to 1.23)
All	Men	80-84	0.35 (-0.48 to 1.18)
All	Men	≥ 85	2.52 (0.98 to 4.09)
All	Men	net_drift	-0.1 (-1.34 to 1.15)
All	Women	20-24	-0.81 (-5.77 to 4.41)
All	Women	25-29	-1.7 (-4.87 to 1.58)
All	Women	30-34	-1.21 (-3.35 to 0.99)
All	Women	35-39	-0.94 (-2.58 to 0.73)
All	Women	40-44	-2.07 (-3.33 to -0.79)
All	Women	45-49	-1.09 (-2.09 to -0.07)
All	Women	50-54	-1.28 (-2.11 to -0.43)
All	Women	55-59	-0.91 (-1.65 to -0.17)
All	Women	60-64	-1.47 (-2.14 to -0.8)
All	Women	65-69	-0.33 (-0.94 to 0.27)
All	Women	70-74	-0.13 (-0.68 to 0.43)
All	Women	75-79	0.19 (-0.38 to 0.76)
All	Women	80-84	0.46 (-0.24 to 1.15)
All	Women	≥ 85	2.58 (1.4 to 3.77)
All	Women	net_drift	-1.18 (-2.65 to 0.3)
Urban	Men	20-24	-1.09 (-7.53 to 5.8)
Urban	Men	25-29	-3.17 (-7.66 to 1.53)
Urban	Men	30-34	-2.74 (-5.89 to 0.52)
Urban	Men	35-39	-1.9 (-4.29 to 0.56)
Urban	Men	40-44	-3.18 (-4.89 to -1.44)
Urban	Men	45-49	-2.17 (-3.42 to -0.91)
Urban	Men	50-54	-1.58 (-2.56 to -0.59)
Urban	Men	55-59	-0.07 (-0.91 to 0.78)
Urban	Men	60-64	0.11 (-0.64 to 0.88)
Urban	Men	65-69	0.97 (0.28 to 1.67)
Urban	Men	70-74	0.12 (-0.52 to 0.77)
Urban	Men	75-79	-0.84 (-1.5 to -0.18)
Urban	Men	80-84	-0.86 (-1.68 to -0.04)
Urban	Men	≥ 85	2.05 (0.54 to 3.58)
Urban	Men	net_drift	-1.48 (-3.21 to 0.28)
Urban	Women	20-24	0.47 (-6.68 to 8.16)
Urban	Women	25-29	-2.92 (-7.3 to 1.68)
Urban	Women	30-34	-1.53 (-4.39 to 1.41)
Urban	Women	35-39	-0.83 (-2.99 to 1.38)
Urban	Women	40-44	-2.24 (-3.87 to -0.59)
Urban	Women	45-49	-2.23 (-3.5 to -0.95)
Urban	Women	50-54	-2.74 (-3.76 to -1.71)
Urban	Women	55-59	-1.83 (-2.72 to -0.93)
Urban	Women	60-64	-1.81 (-2.61 to -1.0)
Urban	Women	65-69	-1.24 (-1.95 to -0.52)
Urban	Women	70-74	-1.82 (-2.46 to -1.17)
Urban	Women	75-79	-1.8 (-2.43 to -1.15)
Urban	Women	80-84	-0.62 (-1.38 to 0.14)
Urban	Women	≥ 85	2.5 (1.22 to 3.79)
Urban	Women	net_drift	-1.48 (-3.43 to 0.5)
Rural	Men	20-24	0.36 (-4.3 to 5.25)
Rural	Men	25-29	-0.08 (-3.24 to 3.19)
Rural	Men	30-34	0.27 (-2.1 to 2.69)
Rural	Men	35-39	-0.03 (-1.88 to 1.86)
Rural	Men	40-44	-1.05 (-2.43 to 0.36)
Rural	Men	45-49	0.22 (-0.87 to 1.32)
Rural	Men	50-54	0.4 (-0.51 to 1.32)
Rural	Men	55-59	0.66 (-0.13 to 1.46)
Rural	Men	60-64	0.01 (-0.7 to 0.72)
Rural	Men	65-69	1.36 (0.7 to 2.02)
Rural	Men	70-74	1.89 (1.25 to 2.54)
Rural	Men	75-79	1.93 (1.23 to 2.64)
Rural	Men	80-84	1.71 (0.74 to 2.7)
Rural	Men	≥ 85	3.21 (1.37 to 5.08)
Rural	Men	net_drift	0.56 (-0.58 to 1.71)
Rural	Women	20-24	-1.28 (-6.14 to 3.83)
Rural	Women	25-29	-1.1 (-4.25 to 2.16)
Rural	Women	30-34	-1.05 (-3.22 to 1.16)
Rural	Women	35-39	-0.98 (-2.65 to 0.73)
Rural	Women	40-44	-1.94 (-3.24 to -0.62)
Rural	Women	45-49	-0.26 (-1.32 to 0.81)
Rural	Women	50-54	-0.23 (-1.13 to 0.67)
Rural	Women	55-59	-0.18 (-0.98 to 0.62)
Rural	Women	60-64	-1.1 (-1.83 to -0.37)
Rural	Women	65-69	0.52 (-0.15 to 1.19)
Rural	Women	70-74	1.35 (0.72 to 1.99)
Rural	Women	75-79	1.93 (1.28 to 2.59)
Rural	Women	80-84	1.64 (0.82 to 2.46)
Rural	Women	≥ 85	2.96 (1.55 to 4.38)
Rural	Women	net_drift	-0.73 (-2.02 to 0.58)
Eastern region	Men	20-24	-1.94 (-7.35 to 3.79)
Eastern region	Men	25-29	-2.45 (-6.14 to 1.39)
Eastern region	Men	30-34	-0.5 (-3.23 to 2.31)
Eastern region	Men	35-39	0.38 (-1.72 to 2.52)
Eastern region	Men	40-44	-1.09 (-2.59 to 0.43)
Eastern region	Men	45-49	-1.42 (-2.52 to -0.3)
Eastern region	Men	50-54	-0.93 (-1.8 to -0.05)
Eastern region	Men	55-59	0.11 (-0.64 to 0.86)
Eastern region	Men	60-64	0.52 (-0.16 to 1.2)
Eastern region	Men	65-69	1.31 (0.69 to 1.94)
Eastern region	Men	70-74	0.88 (0.3 to 1.46)
Eastern region	Men	75-79	0.18 (-0.41 to 0.78)
Eastern region	Men	80-84	0.23 (-0.52 to 0.99)
Eastern region	Men	≥ 85	2.5 (1.16 to 3.85)
Eastern region	Men	net_drift	-0.5 (-1.99 to 1.02)
Eastern region	Women	20-24	0.23 (-6.51 to 7.46)
Eastern region	Women	25-29	-1.38 (-5.61 to 3.05)
Eastern region	Women	30-34	-0.46 (-3.33 to 2.5)
Eastern region	Women	35-39	0.51 (-1.63 to 2.7)
Eastern region	Women	40-44	-1.04 (-2.68 to 0.62)
Eastern region	Women	45-49	-1.6 (-2.87 to -0.31)
Eastern region	Women	50-54	-1.93 (-2.96 to -0.89)
Eastern region	Women	55-59	-1.62 (-2.51 to -0.72)
Eastern region	Women	60-64	-1.29 (-2.1 to -0.47)
Eastern region	Women	65-69	-0.19 (-0.92 to 0.55)
Eastern region	Women	70-74	-0.22 (-0.88 to 0.44)
Eastern region	Women	75-79	-0.28 (-0.92 to 0.37)
Eastern region	Women	80-84	0.2 (-0.55 to 0.96)
Eastern region	Women	≥ 85	3.52 (2.22 to 4.83)
Eastern region	Women	net_drift	-0.98 (-2.82 to 0.89)
Central region	Men	20-24	0.47 (-5.28 to 6.57)
Central region	Men	25-29	0.06 (-3.73 to 4.0)
Central region	Men	30-34	-0.33 (-2.99 to 2.41)
Central region	Men	35-39	-1.48 (-3.55 to 0.64)
Central region	Men	40-44	-2.57 (-4.12 to -0.98)
Central region	Men	45-49	-0.77 (-1.97 to 0.44)
Central region	Men	50-54	-0.34 (-1.33 to 0.66)
Central region	Men	55-59	0.16 (-0.7 to 1.02)
Central region	Men	60-64	-0.71 (-1.48 to 0.07)
Central region	Men	65-69	0.36 (-0.36 to 1.08)
Central region	Men	70-74	0.49 (-0.2 to 1.19)
Central region	Men	75-79	0.34 (-0.41 to 1.1)
Central region	Men	80-84	-0.79 (-1.79 to 0.22)
Central region	Men	≥ 85	0.26 (-1.71 to 2.27)
Central region	Men	net_drift	-0.34 (-1.65 to 0.99)
Central region	Women	20-24	-0.79 (-7.66 to 6.59)
Central region	Women	25-29	-2.81 (-6.99 to 1.57)
Central region	Women	30-34	-1.89 (-4.73 to 1.05)
Central region	Women	35-39	-1.57 (-3.75 to 0.67)
Central region	Women	40-44	-2.18 (-3.86 to -0.48)
Central region	Women	45-49	-1.25 (-2.6 to 0.13)
Central region	Women	50-54	-1.42 (-2.54 to -0.27)
Central region	Women	55-59	-1.2 (-2.2 to -0.19)
Central region	Women	60-64	-2.12 (-3.03 to -1.2)
Central region	Women	65-69	-0.99 (-1.82 to -0.15)
Central region	Women	70-74	-0.54 (-1.31 to 0.24)
Central region	Women	75-79	-0.03 (-0.84 to 0.8)
Central region	Women	80-84	-0.2 (-1.22 to 0.84)
Central region	Women	≥ 85	-0.35 (-2.03 to 1.35)
Central region	Women	net_drift	-1.47 (-3.09 to 0.17)
Western region	Men	20-24	1.1 (-4.09 to 6.58)
Western region	Men	25-29	-0.36 (-4.07 to 3.48)
Western region	Men	30-34	-1.05 (-3.85 to 1.83)
Western region	Men	35-39	-0.82 (-3.03 to 1.45)
Western region	Men	40-44	-1.78 (-3.41 to -0.12)
Western region	Men	45-49	0.65 (-0.68 to 1.99)
Western region	Men	50-54	0.46 (-0.64 to 1.58)
Western region	Men	55-59	0.88 (-0.09 to 1.85)
Western region	Men	60-64	-0.19 (-1.05 to 0.67)
Western region	Men	65-69	1.64 (0.84 to 2.44)
Western region	Men	70-74	2.11 (1.33 to 2.9)
Western region	Men	75-79	1.91 (1.05 to 2.77)
Western region	Men	80-84	2.22 (1.02 to 3.44)
Western region	Men	≥ 85	5.53 (3.14 to 7.97)
Western region	Men	net_drift	0.38 (-0.92 to 1.69)
Western region	Women	20-24	-2.25 (-9.01 to 5.03)
Western region	Women	25-29	-1.07 (-5.73 to 3.82)
Western region	Women	30-34	-1.7 (-4.84 to 1.54)
Western region	Women	35-39	-2.47 (-4.98 to 0.11)
Western region	Women	40-44	-3.59 (-5.52 to -1.61)
Western region	Women	45-49	-0.06 (-1.64 to 1.55)
Western region	Women	50-54	0.02 (-1.34 to 1.4)
Western region	Women	55-59	0.69 (-0.54 to 1.93)
Western region	Women	60-64	-1.06 (-2.16 to 0.05)
Western region	Women	65-69	0.22 (-0.77 to 1.23)
Western region	Women	70-74	0.76 (-0.19 to 1.71)
Western region	Women	75-79	1.69 (0.67 to 2.73)
Western region	Women	80-84	2.18 (0.85 to 3.52)
Western region	Women	≥ 85	4.14 (1.85 to 6.49)
Western region	Women	net_drift	-1.11 (-2.85 to 0.67)

For adult females aged 20 and above, the ASMR of CRC also remained generally stable throughout the study period. However, women aged 85 and above exhibited a consistent annual increase in mortality, with an APC of 2.58% (95% CI: 1.40–3.77%). Further stratified analysis revealed that both urban and rural women aged 70 and above experienced upward trends in mortality, with the highest increases observed among those aged 85 and above. Specifically, the APC was 2.50% (95% CI: 1.22–3.79%) in urban areas and 2.96% (95% CI: 1.55–4.38%) in rural areas. In terms of regional distribution, women aged 85 and above in the eastern region and those aged 75 and above in the western region showed increasing trends. The most significant annual increase was observed in women aged 85 and above in the eastern region, with an APC of 3.52% (95% CI: 2.22–4.83%). No significant trends were found in the central region. In the western region, the greatest increase was again in the 85+ female group, with an APC of 4.14% (95% CI: 1.85–6.49%). In summary, while the overall CRC ASMR among adults aged 20 and above remained stable—suggesting that the population-level disease burden has not markedly worsened—there was a notable upward trend in mortality among older adults, especially elderly men (in urban, rural, eastern, and western regions) and elderly women (also in urban, rural, eastern, and western regions).

## 4 Discussion

Based on national data from the China Disease Surveillance Points (DSP) system from 2004 to 2021, this study identified several important findings. First, overall CRC mortality remained relatively stable during the study period. Second, mortality increased significantly among the oldest age group (≥85 years). Third, clear disparities were observed, with higher mortality among men, urban residents, and populations in the eastern region. Finally, the age-related gaps in sex and urban–rural differences widened progressively with age, with the most pronounced differences observed in the oldest age group. CRC remains one of the leading causes of cancer-related death worldwide, and its disease burden varies substantially across populations and regions. These findings are generally consistent with global epidemiological trends, but also reflect the unique disease distribution characteristics of China.

From 2004 to 2021, CRC mortality among adults aged 20 and above showed an upward trend, particularly among the elderly. This is consistent with previous research findings (11). Part of the increase in mortality can be attributed to known cancer risk factors (2, 12). In recent decades, China’s aging population has steadily grown, contributing to a heavier cancer burden (13). The strong age gradient observed in this study is consistent with the well-established association between advancing age and CRC risk. Studies have shown that the prevalence of CRC is significantly higher among people over 50 compared to younger age groups (14). China is facing a rapid aging process, especially in urban areas (54%) and economically developed eastern regions (39.1%) (15), where the proportion of elderly individuals is higher. Population aging may therefore contribute to the higher mortality observed in these regions.

This study found that the mortality rate of CRC in Chinese men is significantly higher than that in women, a phenomenon consistent with global epidemiological trends (16) and with previous research results in China (5, 17). Previous studies have shown that the incidence and mortality rates of CRC are generally higher in men than in women. These differences are commonly attributed to variations in lifestyle and behavioral risk factors between men and women. Men have higher exposure rates to smoking, drinking, and unhealthy diets. Studies have found that smokers with CRC have a 30%-40% higher mortality rate than non-smokers (14). In 2015, Chinese men consumed over one-third of the world’s cigarettes, while smoking rates among women were much lower, especially among younger generations (18). These behavioral differences may partly explain the higher CRC mortality observed in men.Excessive alcohol consumption has been identified as an established risk factor for CRC. The 2018 report by the World Cancer Research Fund/American Institute for Cancer Research (WCRF/AICR) indicated that higher levels of alcohol intake are associated with increased CRC risk (19). Experimental evidence also supports plausible biological pathways linking alcohol exposure to colorectal carcinogenesis (20, 21).

However, alcohol consumption represents only one component of a broader behavioral and metabolic risk profile. Sex differences in CRC mortality are likely influenced by multiple interrelated factors, including dietary composition, obesity prevalence, metabolic health, and healthcare utilization patterns. In China, men report higher levels of alcohol consumption than women (22), but this behavioral disparity exists within a wider constellation of lifestyle exposures that may collectively contribute to the observed mortality differences.

Urban residents tend to have more Westernized diets, with a greater prevalence of low-fiber and high-fat foods such as red and processed meats. People in urban areas are more likely to consume these foods than those in rural areas, and the harmful metabolic consequences of these foods may be a contributing factor to the progression of CRC (14, 23, 24). Red and processed meats contain heme iron and nitrites, which can be converted into carcinogens in the intestines. High-fat diets alter gut microbiota and increase bile acid secretion, promoting CRC development. In contrast, rural diets are generally higher in grains and vegetables, with greater dietary fiber intake, which may offer some protective effects. However, with the narrowing dietary gap between urban and rural populations in recent years, CRC mortality has gradually increased in rural areas. These patterns suggest that lifestyle and dietary transitions may play a role in the observed urban–rural differences, although causal relationships cannot be established in this study.

Furthermore, sedentary lifestyles and lack of physical activity have been associated with increased CRC risk, particularly among middle-aged and older adults (25). Urban residents are more likely to adopt sedentary lifestyles and have higher prevalence of overweight and obesity (26). Obesity has been linked to CRC through mechanisms such as chronic inflammation and insulin resistance (27–29). These factors may contribute to the higher CRC mortality observed in urban populations. In addition, people with lower socioeconomic status often face higher CRC mortality (24, 30), which may be related to low screening participation and limited access to medical resources.

This study found that CRC mortality in the eastern region of China is significantly higher than in the central and western regions, consistent with previous findings (24, 30, 31). Although the eastern region is more economically developed and has better primary healthcare resources, the CRC mortality rate among the elderly remains high. The eastern region also experiences more pronounced population aging than the other regions (32, 33). Population aging and lifestyle-related risk factors may partly explain the higher CRC mortality observed in this region.

Despite advances in medical technology, the elderly population in western China has experienced a significant annual increase in CRC mortality. In terms of healthcare resource allocation, the western region—particularly rural western areas—suffers from inadequate infrastructure and a shortage of medical professionals (34, 35). This results in low coverage of early CRC screening and limited diagnostic capacity. In addition, health education and disease prevention awareness remain insufficient in some remote areas (36, 37), and geographical barriers reduce access to specialized medical care (38). These structural factors may contribute to regional disparities in CRC mortality.

## 5 Limitations

This study used data from the DSP system to analyze trends in CRC mortality in China. Although the dataset provides nationwide coverage and long-term monitoring, it still has some limitations.

First, although the DSP system has established a nationwide monitoring network through scientific sampling, its coverage of special populations (such as migrant populations and ethnic minority settlements) may still be insufficient. Although the setting of monitoring points takes into account the geographical distribution balance, the coverage depth of extremely remote areas may be limited, which may affect the accurate capture of certain region-specific death patterns. Therefore, some degree of selection bias related to the sampling framework of the DSP system cannot be completely excluded, and the findings may not be fully generalizable to all subpopulations in China.

Second, the system does not routinely collect individual-level risk factor data (such as dietary habits, CRC screening history, family history, etc.), which limits our in-depth analysis of the driving factors behind mortality changes. At the same time, regional differences in medical resource accessibility and treatment norms are not fully reflected in the existing data framework. As a result, residual confounding from unmeasured behavioral, clinical, and socioeconomic factors may remain.

Third, the observed increase in mortality among the oldest age group (≥85 years) may partly reflect the higher burden of comorbidities and potential misclassification of the underlying cause of death in very old individuals. In settings with limited diagnostic capacity, particularly in rural and western regions, cause-of-death attribution may be less precise, which could introduce some degree of reporting bias. Although quality control procedures are implemented in the DSP system, underreporting and misclassification of causes of death may still occur.

Finally, as this study relies on aggregated population-level data, the findings reflect group-level patterns and cannot support individual-level causal inference. Ethnicity-specific analyses, especially relevant in western China, were not feasible due to data limitations.

## 6 Conclusion

The mortality rate of CRC in China shows significant differences among populations and regions. The burden is particularly heavy for men, urban residents, and people in the eastern coastal areas. Strengthening early screening for high-risk groups and optimizing the allocation of regional medical resources are crucial for reducing the burden of CRC.

## Data Availability

The raw data supporting the conclusions of this article will be made available by the authors, without undue reservation.
